# Flux Imbalance Analysis and the Sensitivity of Cellular Growth to Changes in Metabolite Pools

**DOI:** 10.1371/journal.pcbi.1003195

**Published:** 2013-08-29

**Authors:** Ed Reznik, Pankaj Mehta, Daniel Segrè

**Affiliations:** 1 Department of Biomedical Engineering, Boston University, Boston, Massachusetts, United States of America; 2 Department of Physics, Boston University, Boston, Massachusetts, United States of America; 3 Bioinformatics Program, Boston University, Boston, Massachusetts, United States of America; 4 Department of Biology, Boston University, Boston, Massachusetts, United States of America; The Pennsylvania State University, United States of America

## Abstract

Stoichiometric models of metabolism, such as flux balance analysis (FBA), are classically applied to predicting steady state rates - or fluxes - of metabolic reactions in genome-scale metabolic networks. Here we revisit the central assumption of FBA, *i.e.* that intracellular metabolites are at steady state, and show that deviations from flux balance (*i.e.* flux imbalances) are informative of some features of *in vivo* metabolite concentrations. Mathematically, the sensitivity of FBA to these flux imbalances is captured by a native feature of linear optimization, the dual problem, and its corresponding variables, known as shadow prices. First, using recently published data on chemostat growth of *Saccharomyces cerevisae* under different nutrient limitations, we show that shadow prices anticorrelate with experimentally measured degrees of growth limitation of intracellular metabolites. We next hypothesize that metabolites which are limiting for growth (and thus have very negative shadow price) cannot vary dramatically in an uncontrolled way, and must respond rapidly to perturbations. Using a collection of published datasets monitoring the time-dependent metabolomic response of *Escherichia coli* to carbon and nitrogen perturbations, we test this hypothesis and find that metabolites with negative shadow price indeed show lower temporal variation following a perturbation than metabolites with zero shadow price. Finally, we illustrate the broader applicability of flux imbalance analysis to other constraint-based methods. In particular, we explore the biological significance of shadow prices in a constraint-based method for integrating gene expression data with a stoichiometric model. In this case, shadow prices point to metabolites that should rise or drop in concentration in order to increase consistency between flux predictions and gene expression data. In general, these results suggest that the sensitivity of metabolic optima to violations of the steady state constraints carries biologically significant information on the processes that control intracellular metabolites in the cell.

## Author Summary

Cellular metabolism is composed of a complex network of biochemical reactions that convert environmental nutrients into biosynthetic building blocks and energetic currency. Genome-scale mathematical models of metabolic networks focus largely on trying to predict the rates – or fluxes - of these reactions. By assuming that the concentrations of intracellular metabolites are at steady-state (flux balance), and invoking optimality, these constraint-based methods for modeling metabolism have offered abundant insight into how metabolic flux is routed through the cell. Here we ask how cellular growth would respond to deviations from steady state (flux imbalance) of every possible intracellular metabolite. This question can be addressed through a sensitivity analysis inherent to linear optimization theory, known as duality. We show how some features of metabolite concentrations, such as their growth-limitation and their transient response, are captured by this sensitivity analysis. Our results suggest that, in addition to predicting fluxes, stoichiometric models offer a valuable route towards probing the metabolites themselves and their relevance to growth dynamics.

## Introduction

Cells endure relentless variations in intra- and extra-cellular conditions. These perturbations propagate through the cell's metabolic and regulatory networks, leading to a diverse range of interdependent, transient responses in the abundance of metabolites, transcripts, and proteins [1]–[3]. In spite of these changing conditions, cells must efficiently allocate molecular resources through the metabolic network to guarantee homeostasis and enable self-reproduction. Understanding how biochemical pathways and regulatory circuits work together to achieve this robustness remains an open problem with major implications for systems and synthetic biology [4]–[7].

One approach to this question is to use genome-scale, constraint-based models of metabolism (such as flux balance analysis, FBA [8]–[11]). These models rely predominantly on reaction network stoichiometry to provide a scalable, largely parameter-free method for linking individual reaction fluxes with global cellular properties, such as growth. Importantly, constraint-based models frequently assume that the cell has been optimized, through selective pressure and evolution, towards some cellular objective (frequently captured in the biomass flux). The major drawbacks of constraint-based approaches (in contrast to mechanistic models of metabolism [12]) are the incapacity to predict metabolite concentrations and the difficulty of making inferences about the dynamics of the system, though recent efforts have made important contributions in overcoming some of these limitations [13], [14].

Here, we show that some features of the behavior of intracellular metabolites are shaped by the interplay between the stoichiometric architecture of the metabolic network and the nutrient limitations imposed by environmental conditions, as well as the key role of metabolism as the conduit for allocating cellular resources towards growth. This link between structure and function of metabolism is hidden in a largely unappreciated aspect of the solution to flux balance models, namely the dual solution to the associated linear programming (LP) problem [15]. Our main results are threefold. First, we demonstrate how sensitivities to each steady-state constraint in FBA (referred to as shadow prices, and often automatically calculated when solving an FBA problem [16]–[19]) correlate negatively with experimentally quantified degrees of growth-limitation of a metabolite. Second, we show how the growth-limitation of a metabolite (as captured by its shadow price) provides a window onto the temporal response of that metabolite following an environmental perturbation. In particular, by examining a number of time-dependent metabolomics datasets, we observe that metabolites which have large negative shadow prices also exhibit little temporal variability following a perturbation. Third, we examine the broad applicability of shadow prices to other constraint-based approaches to modeling metabolism. We show that, by studying the shadow prices of a constraint-based model that incorporates high-throughput gene expression data, we are able to predict whether an intracellular metabolite accumulates or depletes. Taken together, our results suggest that shadow prices and “flux imbalance analysis” may find quite useful application in probing the behavior of metabolites using constraint-based modeling.

## Results

### Shifting the Focus from Fluxes to Metabolites: Shadow Prices

Flux balance analysis is a method for computing expected reaction rates in complex metabolic networks, and has been described in detail elsewhere [9], [20]. The basic strategy of FBA is to identify steady state metabolic rates (fluxes) that satisfy a set of constraints, and maximize (or minimize) a given objective function. The main constraints are usually (i) mass conservation (or flux balance) at each metabolite node, due to the steady state approximation, and (ii) a set of inequalities associated with limitation of extracellular metabolites and empirical evaluations of irreversibility. Key inequalities are usually imposed on *exchange reactions*, i.e. source/sink reactions mediating the interaction between a cell and its surrounding environment. A canonical FBA calculation can be formally expressed as the following *primal* LP problem:
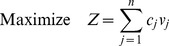
(1)

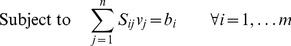
(2)

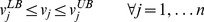
(3)where *S* is the *m* (metabolites) by *n* (reactions) stoichiometric matrix, ***v*** is the vector of metabolic fluxes, ***v^LB^*** is a vector of lower bounds for all fluxes, ***v^UB^*** is a vector of upper bounds for all fluxes, ***b*** is the vector of the rates of accumulation/depletion of each metabolite, and ***c*** is the vector defining the contribution of different fluxes to the objective function.

For intracellular reactions, the right-hand-side coefficients *b_i_* in Eq. (2) are typically assumed to be zero, capturing the assumption that all intracellular metabolites are at steady state. Our analysis is essentially centered on exploring how the cell would respond to deviations from null *b_i_* coefficients. Such a deviation implies a flux imbalance at metabolite *i*, and hence its accumulation or depletion. Importantly, this interpretation of Eq. (2) is not meant as a substitute for the underlying kinetics of the system. Such a flux imbalance may propagate through the metabolic network to influence the optimal value of the objective function *Z*. How can one quantify the sensitivity of the objective function to such flux imbalances? What is the biological significance of these sensitivities?

In fact, every LP calculation can be reformulated in terms of a complementary problem known as the dual problem [15], whose variables (referred to as a *shadow prices*, *λ_i_*) specifically capture the change in the value of the objective function upon a unit change in the right-hand-side of a single constraint (*b_i_*). The general formulation of the dual problem can be found in any linear optimization textbook (e.g. [15]), and its specific formulation for FBA is described in detail in the Methods section. In practice, the shadow prices are typically provided in parallel to the primal variables by any LP solver upon solving Eqs. (1)–(3).

**Figure 1:**
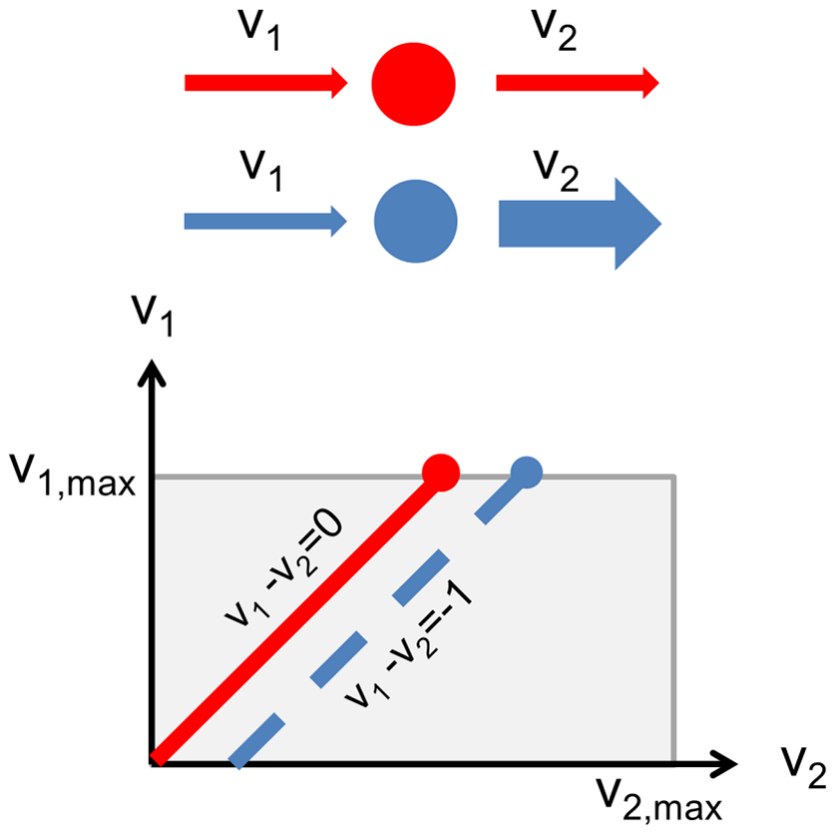
Shadow prices in FBA capture the sensitivity of growth to flux imbalances. Consider the FBA problem with one metabolite and two reactions, formulated as: 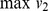, 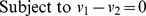; 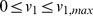; 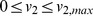. The solid red line indicates the feasible solution space, and the red dot indicates the optimal solution. When the flux balance condition is relaxed and the outgoing flux from *M* is allowed to increase, the feasible space moves to the right (dashed blue line) and the optimal solution increases. Since the objective function increases as the right-hand-side of the flux balance constraint decreases, the metabolite has a negative shadow price. In general for *intracellular* metabolites, negative shadow prices correspond to growth-limiting metabolites.

In analogy with the interpretation of shadow prices in economics and in line with prior work on shadow prices in constraint-based metabolic modeling [16]–[19], FBA's shadow prices estimate the value of each metabolite to the global molecular budget of a growing cell (Figure 1). The interpretation of shadow prices is particularly interesting in the case of the canonical FBA objective function, *i.e.* maximization of the biomass flux (*Z = v_growth_*). In this case, a shadow price corresponds to the change in the biomass flux when one of the intracellular metabolites deviates from steady state. Importantly, if a metabolite has a negative shadow price, this means that allowing additional outflow from this metabolite (so that *b_i_*<0) will increase the maximal value of the biomass flux, implying that this metabolite is *limiting* for the biomass objective (Figure 1). In the remainder of this article, we test the hypothesis that shadow prices correlate with the magnitude of growth-limitation of a metabolite using experimental data, and explore the broader implications of shadow prices in modeling genome-scale metabolism.

### Shadow Prices and Growth Limitation

To explore the connection between shadow prices and growth limitation, we analyzed previously collected experimental data studying the relationship between intracellular metabolite abundances and growth-limitation in *Saccharomyces cerevisae* under continuous culture [21]. For three different conditions (single nutrient limitation on glucose, nitrogen, and phosphate), and two auxotrophic mutants (leucine and uracil) the abundance of intracellular metabolites was quantified for several different dilution (growth) rates.

**Figure 2:**
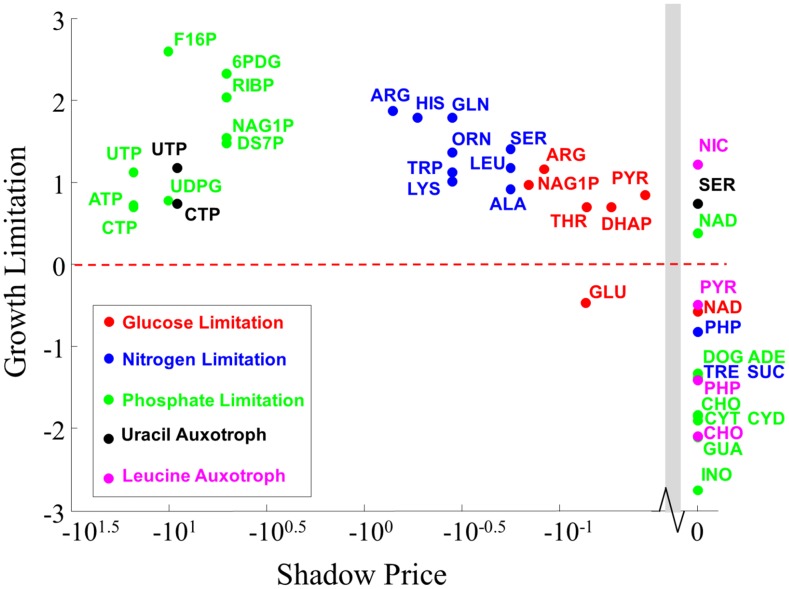
Shadow prices anticorrelate with experimental measurements of growth limitation. Metabolites exhibiting 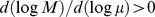 were experimentally determined to be growth-limiting. Growth-limitation 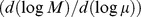 and shadow prices in FBA are significantly anticorrelated under all nutrient limitations from [21]. To make the data more comparable across different nutrient limitations, the data is plotted on a log scale. All points to the left of the grey bar have a shadow price of zero. All correlations for this data (calculated using a linear scale, not the log scale depicted in the Figure) are reported in Table S1. Abbreviations: 6PDG, 6-phospho-d-gluconate; ADE, Adenosine; ALA, Alanine; ARG, Arginine; ATP, ATP; CHO, Choline; CTP, CTP; CYD, Cytidine; CYT, Cytosine; DHAP, Dihydroxyacetone-Phosphate; DOG, Deoxyguanosine; DS7P, D-sedoheptulose-7-phosphate; F16P, Fructose-1,6-bisphosphate; GLN, Glutamine; GLU, Glutamate; GUA, Guanosine; HIS, Histidine; INO, Inosine; LEU, Leucine/isoleucine; LYS, Lysine; NAD, NAD+; NAG1P, N-acetyl-glucosamine-1-phosphate; NIC, Nicotinate; ORN, Ornithine; PHP, Phenylpyruvate; PYR, Pyruvate; RIBP, Ribose-phosphate; SER, Serine; SUC, Sucrose; THR, Threonine; TRE, Trehalose; TRP, Tryptophan; UDPG, UDP-D-glucose; UTP, UTP. For clarity, only cytosolic metabolites from the metabolic model are plotted.

Boer and colleagues [21] showed that the growth limitation of a metabolite could be quantified by measuring the change in metabolite abundance at different, increasing growth rates. In particular, metabolites with relatively low intracellular concentrations which *increased* in abundance as growth rate increased were found to be *growth-limiting*. In contrast, metabolites which relatively high concentration and which *decreased* in concentration as growth rate increased were described as “overflow” metabolites, and were *not growth-limiting*. To understand why we may expect such correlations, we can re-elaborate on the reasoning presented by Boer and colleagues in [21]. As described in [21], we consider the simplest case, where growth is limited by the concentration of a single, growth-limiting nutrient *M*. The dependence of growth on this metabolite can be described by the classical Monod equation:
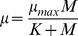
where *K* is the half-saturation constant, 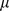 is the growth rate, and 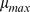 is the maximum growth rate. As we derive in detail in Supplementary Text S1, valuable intuition for the dependence of 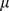 on *M* can be gained by considering the limiting cases *M>>K* and *M<<K*. In the first case, *M* is substantially larger than the half-saturation constant *K*. Then, the growth rate is relatively insensitive to changes in *M*, and it can be treated as non-growth-limiting. By calculating the dependence of *M* on 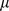 in this limiting case, one finds
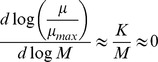
Thus, in this case, we would expect very small correlation between log 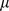 and log *M*. As shown in [21] this correlation can even become negative due to feedback inhibition (corresponding to points below the horizontal red line in Figure 2). In the other limiting case, where *M* is much smaller than *K* (and the growth rate is very sensitive to *M*, so *M* is very growth-limiting)
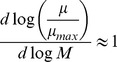
and we expect a positive correlation between 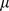 and *M* (corresponding to points above the horizontal red line in Figure 2). The extent of this correlation (together with its sign), constitutes a metabolite-specific metric for growth limitation, and corresponds to the abscissa in the graph of Figure 2. Furthermore, this simple model is readily extendible to cases where many metabolites may simultaneously be limiting for growth rate, as shown in [21].

We compared the growth-limitation measurements for each metabolite identified as significantly growth-limiting or non-growth-limiting/overflow in [21], to the corresponding shadow prices computed through FBA *in silico* experiments when maximizing for biomass production in the yeast model iMM904 [22]. For all natural nutrient limitations, we found an anticorrelation between shadow prices and growth-limitation: growth-limiting metabolites exhibit negative shadow prices, while non-growth-limiting metabolites exhibit small or zero shadow prices (Figure 2). Furthermore, for each individual nutrient condition, the more negative a shadow price was, the more limiting the corresponding metabolite was found to be in the original paper [21]. Although there is little support that the correlation between shadow prices and growth limitation is linear, we report both Spearman (rank-based) and Pearson (linear) correlations. These anticorrelations were strongest for nitrogen (Spearman *ρ* = −0.74, p-value = 2×10^−5^, Pearson *r* = −0.77, p-value = 1×10^−5^) and phosphate limitation (Spearman *ρ* = −0.66, p-value = 5×10^−5^, Pearson *r* = −0.50, p-value = 0.033), where there were substantially more data points (12 and 17 metabolites experimentally identified as significantly growth- or non-growth-limiting in [21], respectively) than for glucose limitation (Spearman *ρ* = −0.59, p-value = 0.008, Pearson *r* = −0.77, p-value = 1×10^−5^, 7 metabolites).

In agreement with [21], the growth-limiting metabolites in each condition reflect the corresponding nutrient limitation. In nitrogen limitation, we found many candidate growth-limiting metabolites, nearly all of which were amino acids. In glucose starvation, we found N-acetyl-glucosamine-1-phosphate (a precursor for protein glycosylation) and arginine to be among the most growth-limiting metabolites (with the most negative shadow price). The main outlier in glucose starvation was glutamate, which had a negative shadow price (*i.e.* predicted to be growth-limiting) even though its concentration was experimentally observed to fall with increasing growth rate. The authors of [21] attributed this peculiar behavior of glutamate to the potential overabundance of nitrogen relative to carbon in extremely carbon-limited environments. Perhaps most interestingly, we found that the largest shadow prices occurred under phosphate limitation (see Figure 2, green dots), in agreement with the large growth-limitation (in comparison to other conditions) reported in [21]. It will be interesting in the future to investigate whether the apparent *strength* of growth-limitation (as quantified by the magnitude of the shadow price) plays a role in the extent to which these metabolites regulate the rates of enzymatic reactions.

We repeated the statistical analyses above for two “lumped” datasets containing data from (i) all three natural nutrient limitation conditions (glucose, nitrogen, and phosphate limitation; Spearman *ρ* = −0.87, p-value 2×10^−8^, Pearson *r* = −0.69, p-value = 8×10^−5^) and (ii) all three nutrient limitation conditions, together with auxotrophies (Spearman *ρ* = −0.70, p-value = 2×10^−13^, Pearson *r* = −0.28, p-value = 0.006). The results also remained valid when we only considered cytosolic metabolites, rather than metabolites from all compartments (see Table S1 and Dataset S1). Finally, we assessed whether the sign of a shadow price (*i.e.* either zero or negative) could be used as a predictive binary classifier for whether a metabolite is growth-limiting or non-growth-limiting. To do so, we calculated the Matthews Correlation Coefficient (MCC) [23], a standard measure for the performance of a binary classifier. We found statistically significant agreement between the sign of a shadow price and its classification as growth-/non-growth-limiting, both when using metabolites from all compartments (MCC 0.61, p-value = 5×10^−8^) and only cytosolic metabolites (MCC 0.81, p-value = 1×10^−7^).

An important question in the above analysis, and in the calculation of shadow prices in general, is whether the possible alternative optima in the FBA optimization problem could give rise to degenerate shadow prices, and hence ambiguity in the comparison with experimental data. As described in detail in the Methods, we addressed this issue by recalculating each shadow price in a brute force way, *i.e.* by solving two additional LP problems where the right-hand-side of each steady-state constraint (*b_i_* in Eq. (2)) is incremented/decremented by a small amount (as explored before in a different context [24] and in detail in the Methods). The shadow prices obtained from solving these two problems correspond to manual (*i.e.* not obtained automatically from the LP solver upon solving the primal) re-calculations of the sensitivity of the objective function to deviations from each steady-state constraint. We then compared the incremental shadow price, decremental shadow price, and the shadow price obtained directly from the LP solver, and found no instances of degeneracy in our shadow price calculations.

Our results so far indicate, in line with our intuition and with prior work on duality in FBA [17], [18], that shadow prices may serve as quantitative measures of the sensitivity of growth rate to the abundance of an intracellular metabolite. In the next section, we investigate whether this sensitivity has implications for the transient dynamics of growth-limiting metabolites following a perturbation.

### Shadow Prices and Metabolic Dynamics

Given the metabolite-specific associations between shadow prices and growth-limitation, we decided to investigate whether shadow prices could also aid in understanding other features of intracellular metabolites. In particular, we reasoned that if a metabolite is truly growth-limiting, then its concentration in the cell should be tightly controlled. If, in contrast, a growth-limiting metabolite's concentration is allowed to fluctuate or vary uncontrollably, this temporal variability would eventually propagate to growth rate and have potentially deleterious consequences. Our reasoning was further bolstered by recent studies of the metabolic response of *Escherichia coli* to sudden perturbations which demonstrated that the growth rate of cells responds remarkably quickly to changes in environmental conditions. In two experiments [25], [26], it was shown that a sudden change in substrate availability in the environmental media led to a rapid change in the growth rate. In [25], a pulse of glucose to a glucose-limited chemostat culture of *E. coli* lead to a 3.7-fold increase in growth rate less than a minute. Similar results were observed in [26] for pulses of pyruvate and succinate.

Based on our reasoning and on the two studies in [25], [26], we hypothesized that growth-limiting metabolites (with very negative shadow prices) should exhibit very little temporal variation in their concentrations in response to perturbations. In contrast, metabolites exhibiting large temporal variation should not be growth-limiting (and have small or zero shadow price). We tested this prediction using multiple time-course metabolomics datasets for *E. coli* for different glucose and nitrogen perturbations [27], [28]. We elected to use these datasets because they contained information for a large number of metabolites (∼70 unique compounds), enabling us to obtain reasonable statistical power. For each dataset, we calculated the temporal variation of a metabolite across the time course following the perturbation, using the coefficient of variation (*CV*, the standard deviation of the time series, divided by its mean; see Methods for more details). Thus, a very large temporal variation corresponded to a circumstance when a metabolite's concentration changed substantially following a perturbation, and a small temporal variation indicated that a metabolite's concentration remained relatively constant post-perturbation. After calculating the temporal variation for each metabolite, we computed the metabolite shadow prices using FBA (see Methods).

**Figure 3:**
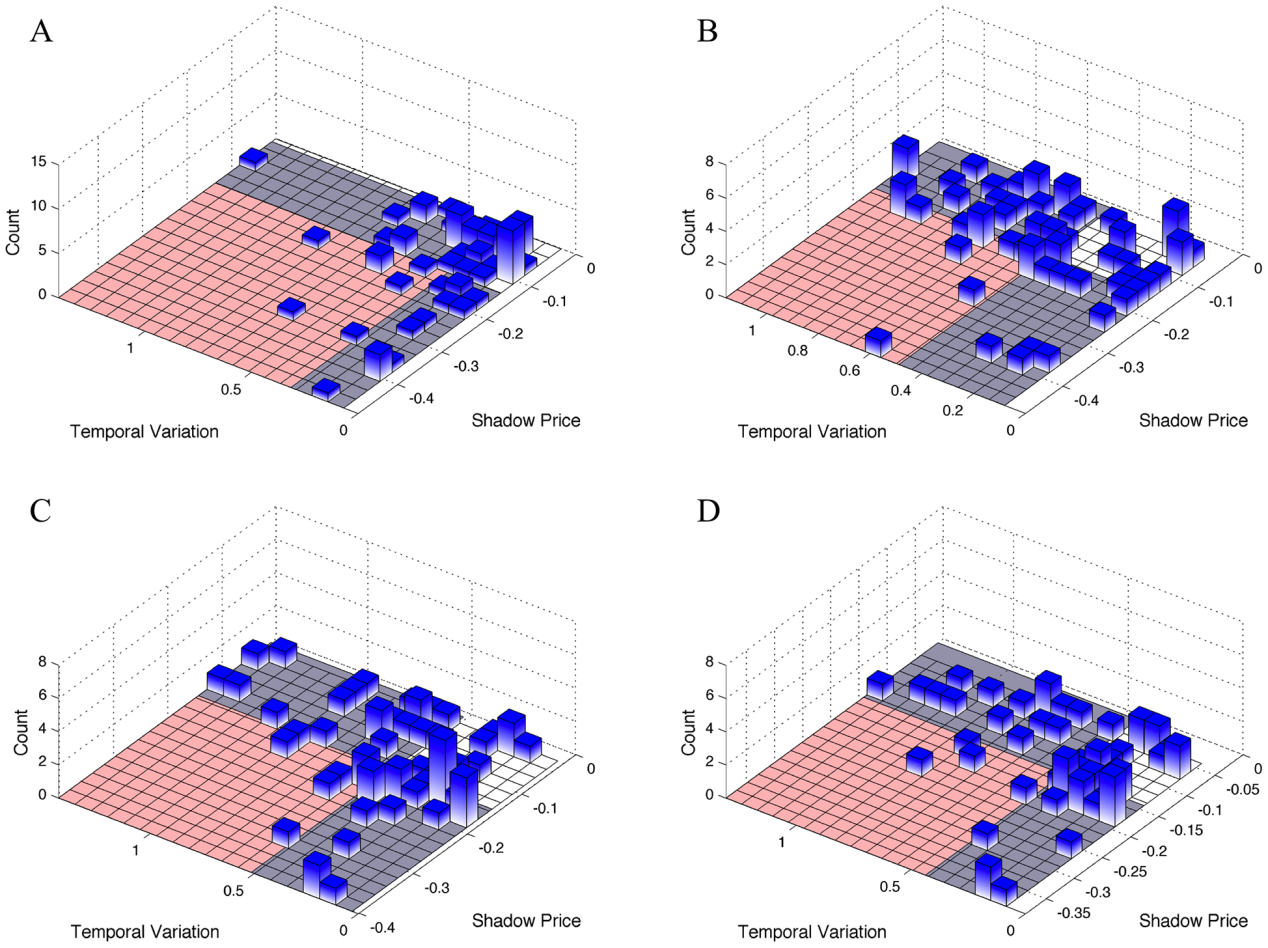
Shadow prices correlate with temporal variation in metabolite abundance in *E.* *coli*. The height of each bar represents the number of individual metabolites that fall within a bin. Boundaries between the blue and red regions in each panel correspond to the mean values of shadow prices and temporal variation, respectively. We expect that metabolites with negative shadow prices should have small temporal variation, while metabolites with large temporal variation should have small or zero shadow prices (gray regions). Furthermore, metabolites should not exhibit large temporal variation if they have large negative shadow prices (red region). Bars tend not to fall in the red regions (as quantified statistically, see reported p-values, Table S2) highlighting the capacity of shadow prices to capture features of metabolite dynamics. Subplots correspond to different experimental conditions: (**A**) nitrogen upshift (**B**) glucose starvation, (**C**) acetate limitation, and (**D**) glycerol limitation.

The results of our analysis are shown in Figure 3. In agreement with our expectations, shadow prices were found to be correlated with temporal variation in the five perturbations we studied. Metabolites with very large, negative shadow prices (and thus very limiting for biomass production) showed little temporal variation. Conversely, metabolites with the largest temporal variation were found to have comparatively smaller shadow prices. We again report both Spearman and Pearson correlations, although there is no *a priori* reason to expect linear correlations. The correlations were statistically significant for nitrogen upshift (Spearman *ρ* = 0.26, p-value = 0.02, Pearson *r* = 0.22, p-value = 0.04), as well as the 4 different carbon perturbations (glucose starvation, Spearman *ρ* = 0.21, p-value = 0.05, Pearson *r* = 0.21, p-value = 0.045; acetate limitation, Spearman *ρ* = 0.37, p-value = 0.002, Pearson *r* = 0.36, p-value = 0.002; succinate limitation, Spearman *ρ* = 0.23, p-value = 0.04, Pearson *r* = 0.18, p-value = 0.08, and glycerol limitation, Spearman *ρ* = 0.32, p-value = 0.007, Pearson *r* = 0.33, p-value = 0.006). These correlations were further substantiated using non-parametric permutation tests, described in the Methods, with results detailed in Table S2.

Despite these statistically significant correlations, a number of outliers (*i.e.*, metabolites with relatively large, negative shadow prices and high temporal variation) appeared in our results. Among the outliers under glucose limitation (Figure 3B), the most notable were cyclic AMP (a signaling molecule) and acetyl-CoA. More interestingly, in both acetate and glycerol limitation, a repeated outlier was fructose 1,6-bisphosphate (FBP). This metabolite was highlighted in one of the two papers from which we obtained the time-series data [28]. As the authors showed there, upon a sudden switch from glucose medium to either no carbon, acetate, succinate, or glycerol, the concentration of FBP dropped suddenly by 15- to 30-fold. This sudden drop in FBP, coupled with its role as an allosteric activator of PEP carboxylase, resulted in the buildup of PEP. This buildup enabled fast uptake of glucose when it re-appears in the media, where it is used as a phosphate donor for the import of glucose. Furthermore, FBP was recently identified as a candidate “flux sensor,” *i.e.* a metabolite whose concentration may change in linear proportion to the flux through glycolysis, via its role as an activator of pyruvate kinase [29]. Thus, the aberrant behavior of FBP (a negative shadow price, but high temporal variation) may be related to its key role in affecting *E. coli'*s response to glucose starvation and carbon limitation through allosteric regulation.

**Figure 4:**
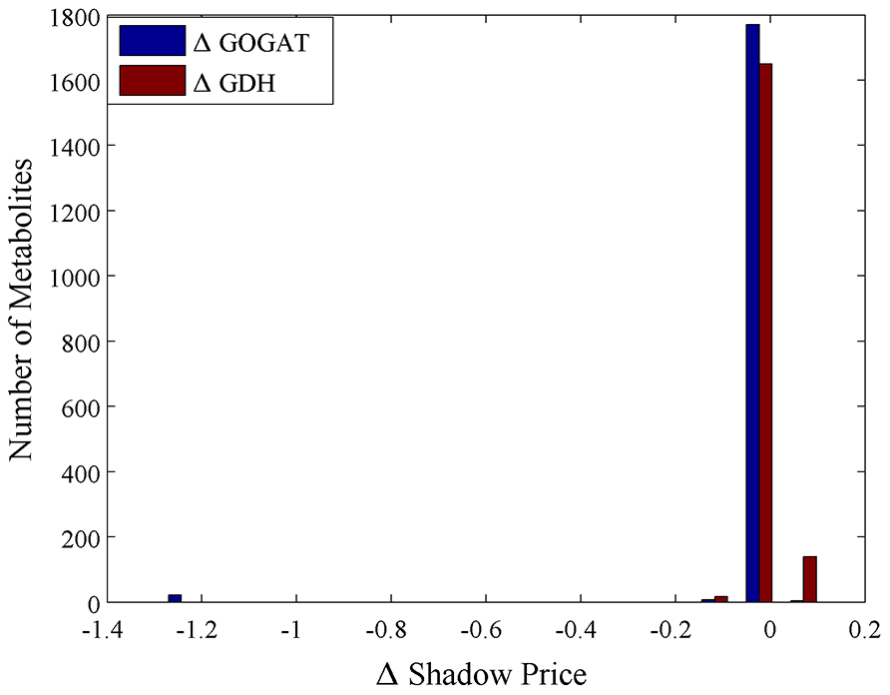
FBA shadow price analysis in knockout strains. Changes in shadow price between the wild-type strain and two knockout strains in *E. coli* following nitrogen upshift. In comparison to the wild-type, only the ΔGOGAT contains any metabolites which are substantially more growth-limiting.

To further corroborate our findings, we tested whether the differential dynamic behavior of mutant knockout strains could be captured through our analysis. We used additional metabolite time series available for the wild type and two knockout strains (ΔGOGAT and ΔGDH) of *E. coli* following nitrogen upshift in [27]. We replicated these knockouts *in silico*, and calculated the shadow prices. We performed two different analyses on this dataset: first, we looked broadly at the changes in shadow prices (from wild-type to knockout) for each of the two knockouts. As illustrated in Figure 4, we found that for the ΔGOGAT strain, 22 metabolites showed a significant drop in shadow price, decreasing by a magnitude greater than one (*i.e.* becoming more growth-limiting). The most drastic changes were found for lipids and precursors, like undecaprenyl phosphate, and UDP-D-glucoronate, both of which showed a drop in shadow price of 1.29. In contrast, the ΔGDH knockout featured no metabolites with a substantial (greater than 0.1) drop in shadow price. This absence of new growth-limiting metabolites in ΔGDH is consistent with the observation in [27] that the GS/GOGAT pathway dominates over GDH in nitrogen limitation. Interestingly, a subset of eight metabolites, all corresponding to glycolipids, showed a substantial *increase* in shadow price in ΔGDH (corresponding to a relaxation in growth-limitation). Thus, while the “growth-limitation landscape” of the ΔGDH mutant, characterized by the most growth-limiting metabolites in the model, seemed relatively similar to that of the wild-type, the ΔGOGAT strain displayed substantially different growth limitations.

Second, we tried to recapitulate the primary qualitative finding of the knockout study from [27]: that glutamine exhibited a substantial drop in temporal variation in ΔGOGAT in comparison to the wild-type and ΔGDH strains (from a sudden increase and then return to steady state in wild type and ΔGDH strains to nearly no response in ΔGOGAT). When comparing the shadow prices of glutamine across the different strains, the shadow price of glutamine dropped from 0 to −0.08, in both ΔGDH and ΔGOGAT. While the shadow prices of other metabolites tracked in the knockout experiments changed as well, glutamine exhibited the largest drop. Thus, despite the fact that an alternative pathway for nitrogen assimilation was present in each knockout strain, the knockout of either GDH or GOGAT led to an increase in growth-limitation of glutamine. This drop in shadow price was in qualitative agreement with the experimentally observed drop in temporal variation in the knockout strains (from 0.85 in wild-type to 0.78 in ΔGDH and 0.32 in ΔGOGAT).

Thus, the shadow prices associated with individual intracellular metabolites provide information not only about the extent to which each metabolite is limiting for growth, but also about its overall temporal variation following a perturbation. Importantly, in the current framework, shadow prices do not provide quantitative predictions about the speed at which metabolites respond, or the new steady-state concentrations they reach. Hence, shadow price analysis should not be treated as a substitute to explicit predictions from kinetics. While our results seem to hold across different experiments in *E. coli* (*i.e.* different nutrient limitations and genetic modifications, albeit in a noisy manner), its general validity and mechanistic basis across different organisms and types of perturbations will require further scrutiny and will be an important aspect of future work. In particular, the availability of specific mechanistic models for the metabolic response to perturbations, coupled with higher temporal resolution data, would allow one to obtain more precise estimates of temporal variability, and hence better quantitative comparisons with shadow prices.

### Shadow Prices and Gene Expression Data

**Figure 5:**
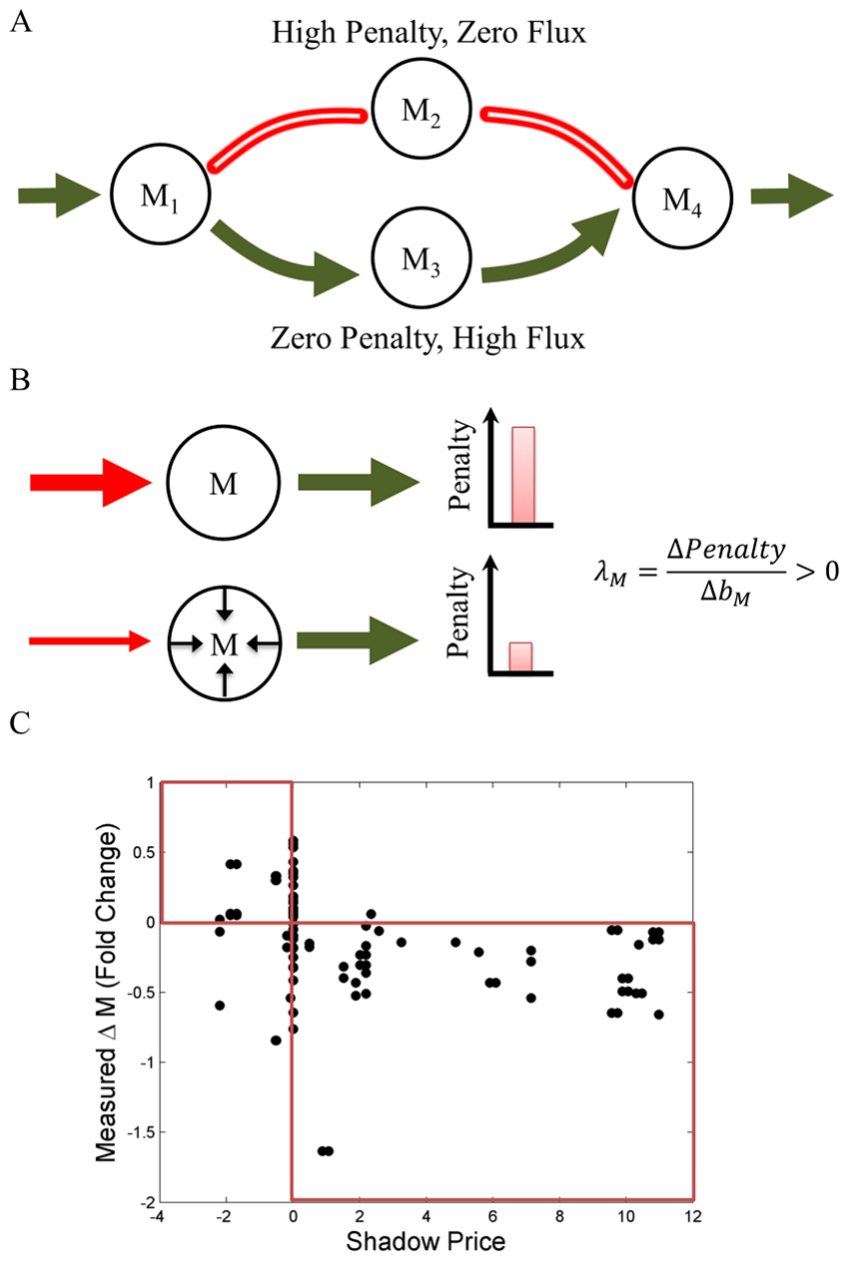
In a constraint-based method that integrates gene expression (GIMME/TEAM), shadow prices predict the direction of changes in metabolite abundance. (**A**) Schematic of the GIMME/TEAM algorithm. Enzymes whose constituent genes show very low expression (red) are penalized. Then, a flux distribution is identified with the lowest total penalty (in this case, the alternative pathway with high expression, colored in green). (**B**) Schematic of the interpretation of shadow prices in TEAM. Consider a situation in which, at steady-state, a reaction with low gene expression (red, high penalty) is inferred by the model to carry a high flux, leading to a high penalty. When the metabolite is allowed to deviate from steady-state by lowering the flux through the highly penalized reaction, the penalty predicted by TEAM falls. The shadow price *λ_M_* for this metabolite, whose concentration is predicted to be decreasing, is thus positive. (**C**) Shadow prices predicted by TEAM and observed changes in metabolite abundance are significantly negatively correlated. A threshold of *θ* = 0.88 was used, although other values of *θ* yielded similar results (SI Figure S1). Changes in metabolite abundance were calculated using measurements between hours 10 and 11 in [33] where acetate was observed to be secreted from the cell [32]. Expression data used as input to TEAM is taken from hour 35 of [32]. Both time points correspond to the same phase in the metabolic cycle of yeast, during the end of the oxidative and beginning of the reductive/building phase.

So far, we have corroborated the notion that shadow prices are indicative of growth limitation, and demonstrated that shadow prices are even more broadly related to metabolite dynamic variability. As described above and in the Methods, shadow prices are dependent on the underlying stoichiometric model, and the specific environmental conditions. Correspondingly, in the analysis shown up to now, we have explored the relevance of shadow prices across different conditions (different nutrient limitations and genetic modifications). There is, however, a third feature that shadow prices crucially depend on, *i.e.* the specific objective function used in the FBA optimization. Does the analysis of shadow prices have a meaning and an application for stoichiometric problems with radically different objective functions, or is it biologically interpretable only for the growth maximization objective? To answer this question, we decided to explore the significance of shadow prices in a recently proposed optimization problem aimed at identifying genome-scale fluxes that minimize the inconsistency relative to a given set of gene expression data. This approach, pioneered with the GIMME algorithm [30], and recently re-elaborated in the time-dependent TEAM method [31], is a way of integrating gene expression data with stoichiometric models of metabolism, in order to obtain better predictions and understanding of cellular physiology. Instead of maximizing growth, GIMME and TEAM minimize the conflict between gene expression data and flux predictions using a penalty score (see Methods). In particular, fluxes whose corresponding gene(s) exhibit low expression are penalized (Figure 5A) in proportion to how much lower the gene expression is, relative to a given gene-specific threshold (see [31] and Methods). The cumulative penalty obtained from all these costs (termed the *Inconsistency Score, IS*) is minimized across the entire metabolic network [30], [31]. This problem can be solved again using linear programming, in analogy to the FBA problem illustrated above:
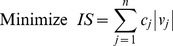
(4)

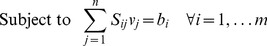
(5)

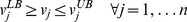
(6)

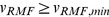
(7)where ***c*** is a vector of reaction penalties, and the reaction flux *v_RMF_* is a *required metabolic functionality* (RMF), some minimal, user-defined metabolic behavior which the model must reproduce (for example, growth at a minimal rate or the secretion of a metabolite). One of the reasons the RMF constraint is imposed is to avoid the trivial solution ***v*** = **0**.

As depicted in Figure 5B, shadow prices in TEAM have a different interpretation from shadow prices in FBA. A shadow price in TEAM is defined as *the change in the inconsistency score* IS *when the steady-state constraint on one metabolite b_i_ deviates from zero*. It is reasonable to assume that some portion of the inconsistency between experimentally measured gene expression and TEAM's flux predictions is the result of imposing the steady state assumption in our model, while a metabolite may be truly accumulating or depleting during certain time intervals in the experiment. Allowing such a metabolite to violate the flux balance condition (either accumulate or deplete) should lower the inconsistency score. Then, if a metabolite's abundance is *decreasing*, we should expect the shadow price to be *positive* (Shadow Price = negative change in *IS*/negative change in abundance). Conversely, if the metabolite's abundance is *increasing*, then we should expect the shadow price to be *negative* (Shadow Price = negative change in *IS*/positive change in abundance). As illustrated in Figure 5B, TEAM's shadow prices should thus be informative of the direction of changes in metabolite abundance: metabolites with positive (negative) shadow price are expected to decrease (increase) in abundance.

To test whether TEAM's shadow prices indeed could predict changes in intracellular metabolite abundances, we re-analyzed a transcriptomics [32] and metabolomics [33] dataset for the yeast metabolic cycle, previously integrated in FBA using TEAM (see [31]). Our analysis (see Methods for details) showed a significant anticorrelation between TEAM's shadow prices and experimentally measured changes in metabolite abundance (Spearman *ρ* = −0.41, p-value = 9×10^−6^; Pearson *r* −0.31, p-value = 8×10^−4^; Figure 5C). Notably, if we only consider those metabolites for which TEAM reported a nonzero shadow price, we correctly identify the direction of change (*e.g.* increase or decrease) in 55 of 63 metabolites in the dataset. We used this data in combination with the Matthews Correlation Coefficient (used earlier to analyze data from Figure 2) as a measure of how well the sign of TEAM's shadow prices can be used to predict the accumulation/depletion of a metabolite. We found that the sign of the shadow price was indeed a good predictor of the direction of change of a metabolite's concentration (MCC 0.68, p-value 7×10^−8^). Interestingly, among the incorrect predictions, many were for amino acids (methionine, ornithine, proline). The failure of TEAM's shadow prices to predict changes in abundance for these compounds suggests that inconsistency with gene-expression data in pathways utilizing these metabolites may not be due to flux imbalances, and may instead indicate that other regulatory mechanisms are at play.

Using the same sensitivity analysis developed in [31] and discussed in the Methods, we furthermore confirmed that the shadow price results reported above were insensitive to changes of the primary free parameter of TEAM, *θ* (a measure for how high to set each gene's penalty threshold), within the range *θ* = 0.50–0.73 and *θ* = 0.78–0.88. This range of thresholds is substantially larger than the range of *θ*'s found to accurately recapitulate experimental data in our studies of *Shewanella oneidensis* using TEAM (*θ* = 0.65 to *θ* = 0.72) [31], suggesting our results here are robust to variations in *θ*. Thus, our analysis of flux imbalances in TEAM, a constraint-based approach based on an objective function radically different from the classical growth maximization of FBA, reveals that shadow prices have useful applications beyond conventional flux balance methods.

## Discussion

Constraint-based stoichiometric models of metabolism have become a widely used approach for characterizing and predicting cellular metabolic states [10]. The notion that steady-state constraints and a cell-level objective function provide an approximate quantitative understanding of the behavior of a population of cells has been subjected to experimental testing, and discussed at length in the literature [8], [11], [34]. Yet, other more subtle aspects of stoichiometric modeling, such as the potential power of shadow prices, had not been directly tested. Nor had the idea of flux imbalance been pursued as a link between the sensitivity analysis of FBA and the dynamics of metabolite pools.

The results we have presented may seem at first glance surprising. How can a steady state solution convey information about the dynamical changes of metabolite pools? The answer is that flux balance models are not simply steady state solutions to a dynamical system. Rather, they use constraints and optimality to predict how a cell should allocate its resources for maximal efficiency, given the underlying network architecture. It would be tempting to make the leap of inferring that the architecture itself truly constrains the dynamics, independent of parameters and regulation. Rather, we suggest that the stoichiometric architecture may dictate how regulation should evolve to guarantee robustness to temporary variations in the intracellular milieu. If the cell cannot allow itself to accumulate or deplete certain metabolites, without incurring a substantial penalty to growth, then the response to variations in these metabolite pools should be swift. This suggests that quick allosteric and post-translational metabolite-induced regulatory feedback should control the stability of these pools [35], [36] and highlights the role the growth process itself may play in providing immediate feedback on metabolite pools by virtue of growth limitation [37]. Thus, we expect that an important challenge for future work will be examining our findings in light of newly reconstructed atlases of metabolic regulatory mechanisms [36].

A subtle but potentially important aspect of shadow prices and their biological interpretation in metabolic network models is the fact that they are defined only over a certain range, as dictated by the structure of the feasible space. These ranges capture how large a perturbation can be before the genome-scale optimal flux distribution changes sharply (*i.e.* by moving to a different corner of the feasible space). In future research, it would be interesting to directly assess the potential existence of such discontinuities in the dynamical behavior of a perturbed metabolic network. In addition, the magnitude of the range of validity of a shadow price may be thought of as an additional tolerance metric for each individual metabolite, conveying the scale beyond which its response to a perturbation becomes unpredictable. Future models may test whether the extent to which a metabolite is regulated depends both on its shadow price, as well as this tolerance to large perturbations.

The sensitivity of cells to variations in specific metabolite pools suggests a novel, metabolite-centric route towards the computational prediction of drug targets, *e.g.* for selectively affecting microbial pathogens or cancer cells. In addition to seeking enzyme gene deletions as a way to impair specific metabolic pathways [38], one could instead impair the regulatory mechanism stabilizing metabolite pools to which growth is particularly sensitive. Notably, the shadow prices automatically generated upon solving the FBA problem would directly provide a prioritization list of the most sensitive target metabolites. It will be interesting to relate the metabolite-centric information obtained from shadow prices to prior quantifications of the importance of metabolites based on their producibility upon gene deletions [39] and on the sum of all incoming or outgoing fluxes around them [40]. Furthermore, one could consider how *lethal* gene deletions/perturbations, which often result in infeasible models for which shadow prices are not immediately available, can be treated using our framework.

Another prospect for future studies will be to evaluate whether shadow prices may shed light on the interplay between evolution, regulation, and the sub-optimal behavior of cells. While most stoichiometric models still use maximization of growth as the central objective, a number of studies have suggested specific applications of alternative objectives. These include the Minimization Of Metabolic Adjustment [41] (or its recent more robust variant, Minimization of Metabolites Balance [42], based on metabolite turnovers instead of fluxes) for describing the cellular phenotypes arising upon genetic perturbations prior to further regulatory or evolutionary optimization, and multi-objective Pareto optimality for studying how cells may sacrifice optimal growth in favor of tradeoff solutions [43]. In the same spirit as our *ad hoc* interpretation and analysis of TEAM's shadow prices, sensitivity of these optimization problems to their respective constraints may offer further insights into the cellular response to perturbations.

Additionally, upon availability of comprehensive data on intracellular metabolite concentrations at multiple time steps, one could envisage implementing stoichiometric models that use explicit flux imbalances (rates of accumulation/depletion) as inputs to the constraint-based model. For example, our shadow price analysis with TEAM is readily extendible to cases where the rate of accumulation/depletion is known for one subset of metabolites, but unknown for another set (*e.g.* for metabolites for which precise intracellular measurements are technically difficult). In such circumstances, for every metabolite for which appropriate data is available, the right-hand-side of the corresponding steady-state constraint (*e.g. b_i_*) could be adjusted accordingly.

Finally, while the notion of flux imbalance analysis is not the first to bridge between the worlds of stoichiometry and metabolic dynamics [14], [44], it is the first to use a genome-scale modeling approach to make inferences about the qualitative response of metabolite concentrations to a perturbation. We do not know the mechanism which induces relatively fast changes in growth-limiting metabolites, when compared to non-growth-limiting metabolites. Indeed, an exciting prospect for future work will be bridging our findings with well-established schools of metabolic theory, including metabolic control analysis [44], biochemical systems theory [45], and structural kinetic modeling [46], [47]. Compellingly, the dual of the FBA problem has also been suggested to constitute a window onto the thermodynamics of biochemical networks, with potential implications for understanding the energetics of metabolism [19]. Unifying these distinct threads, which independently derive dynamic and energetic meaning from the same mathematical framework, seems a worthwhile direction for future efforts.

## Methods

### The Dual Problem to FBA and Shadow Prices

We offer here a simple derivation of the dual problem to flux balance analysis. We begin by posing the primal FBA problem
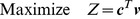
(8)

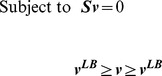
where ***c***, ***v***, ***v^LB^***, and ***v^UB^*** are vectors of length *n*, and ***S*** is the *m*×*n* stoichiometric matrix. For clarity and in contrast to the main text, we have formulated the FBA problem in vector notation (including inequalities, to be interpreted component-wise). We associate with each set of constraints in the primal problem a single set of dual variables. For the steady state constraints, we assign variables 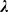 (a vector of length *m*, the shadow prices which we use throughout this work), for the constraints on the lower bounds of each flux, we assign variables 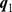 (a vector of length *n*), and for the constraints on the upper bounds of each flux, we assign variables 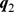 (a vector of length *n*). Then, following any standard text on linear optimization (*e.g.* [15]) one can obtain from Eq. (8) the dual problem
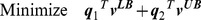
(9)

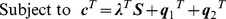


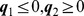



### Alternate and Degenerate Shadow Prices

We implemented a number of measures to ensure that each shadow price used in our calculations was accurate and meaningful. In particular, we validated that the shadow prices obtained directly from the LP solver could not take on different values depending on whether a metabolite was accumulating or depleting (*i.e.* that the shadow price was not degenerate, described below). To do so, we used brute-force techniques to validate that each shadow price reported by the solver was indeed the sensitivity of the objective function to each steady-state metabolite constraint. This process thus simultaneously helped ensure that our results were robust to alternative dual optima.

In addition to the primal solution (optimal fluxes), the Gurobi LP solver provides the corresponding dual solution to the FBA problem. The dual solution contains (i) the shadow price value relative to each metabolite steady-state constraint and (ii) the upper (***G^+^***) and lower (***G^−^***) bounds for which these shadow prices are valid. These bounds indicate the maximum that the right hand side of each constraint may be perturbed while still maintaining the validity of each shadow price. First, we ensured that any calculated shadow prices had non-zero ranges of validity (**range** = ***G^+^−G^−^***). Any shadow prices which did not exhibit a minimal range *ε*_range_ = 10^−6^ were discarded. Other tested values of *ε*_range_ in the range 10^−3^ to 10^−6^ led to qualitatively identical results.

Second, we ensured that alternate optimal solutions [48] did not impact the dual solution. Prior work has reported that degenerate solutions can lead to differences between the incremental shadow price λ^+^ (the change in the objective function when the right-hand-side of a constraint is *increased*) and the decremental shadow price λ^−^ (the change in the objective function when the right-hand-side of a constraint is *decreased*) [24]. To ensure that this did not affect our shadow price calculations, we manually re-calculated the incremental and decremental shadow price for each metabolite for which we had experimental concentration data (indexed by *i_test_*) in the model using a perturbation procedure. This calculation was implemented by solving the two following optimization problems, in which the steady state constraint is positively or negatively violated at each individual metabolite:
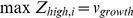
(10)

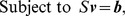


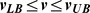


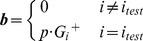


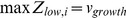
(11)

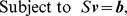


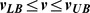


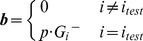
Here, the parameter *p* modulates how large we allow a steady-state constraint to be violated, while remaining in the range [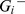, 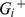] (where the 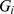 are those defined above with reference to the range of each shadow price). Thus, 0*<p<*1. We used *p* = 0.2, although other choices of *p* yielded identical results (we tried *p* = 0.5 and *p* = 0.9). Upon solving the above optimization problems, the incremental and decremental shadow prices can be computed as the changes in the objective relative to the changes in the right-hand side terms, i.e., respectively:
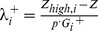
(12)

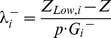
(13)where *Z* is the solution to the regular FBA problem (i.e. the one without perturbations of the right-hand sides). We then ensured that the shadow price obtained from the solver deviated from 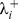 and 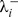 less than the error tolerance of the solver. In many cases, one of 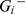 or 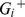 was equal to zero (*i.e.* the shadow price was only valid when perturbing in one direction). In these cases, we only manually calculated the shadow price corresponding to the valid direction. It is important to note that 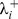 and 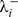 are obtained through brute-force re-calculation of the shadow prices obtained directly from the solver. While they are laborious, they enable us to ensure that degenerate solutions do not adversely affect our results.

In order to facilitate the implementation of degeneracy checking of shadow prices, we have provided the pseudocode below:

CHECK_DEGENERACY(S,LowerBound,UpperBound,Objective)

1 # *Run FBA and obtain four outputs: the optimal flux vector, the shadow prices for each metabolite, the incremental range over which each shadow price is valid, the decremental range over which each shadow price is valid, and the optimal value*

2 [Flux SP SPUpRange SPDownRange OptVal] = ***Run_FBA***(S, LowerBound,UpperBound,Objective, RHSConstraints)

3 p = 0.5 # *p can take value between 0 and 1*

4 # *For every metabolite, check for degeneracy in the shadow price of the metabolite by changing one of the steady-state constraints from zero to a non-zero value within the range of validity*

5 **for** i = 1…number of metabolites

6  **if** SPUpRange(i) >0: # *if we can perturb up*

7   RHSConstraintsPlus = RHSConstraints # *Use a temporary variable*

8   RHSConstraintsPlus(i) = SPUpRange(i)*p # *Change one constraint*

9   *# NEXT: Solve FBA with new constraint (incremental shadow price)*

10 [FluxPlus SPPlus SPUpRangePlus SPDownRangePlus OptValPlus] = ***Run_FBA***(S, LowerBound,UpperBound,Objective, RHSConstraintsPlus)

11  **end** #*end if*

12  **if** SPDownRange(i) <0: # *if we can perturb down*

13   RHSConstraintsMinus = RHSConstraints;

14   RHSConstraintsMinus(i) = SPDownRange(i)*p;

15   *# NEXT: Solve FBA with new constraint (decremental shadow price)*

16   [FluxMinus SPMinus SPUpRangeMinus SPDownRangeMinus OptValMinus] = ***Run_FBA***(S, LowerBound,UpperBound,Objective, RHSConstraintsMinus)

17  **end** # *end if*

18  # *Compare manually calculated shadow prices (if they exist) to solver's*

19  SPPlus(i) = (OptValPlus – OptVal)/SPPlusRange(i)*p

20  SPMinus(i) = (OptValMinus – OptVal)/SPMinusRange(i)*p

21  **if** |SPPlus(i) – SP(i)|>tolerance OR |SPMinus(i) – SP(i)|>tolerance

22   return ERROR # *There is a degenerate shadow price*

23  **end** #*end if*

24 **end** # *end for*

### Software for the Solution of the FBA Primal Problem

In this work, all optimization problems were solved using the Gurobi optimization software [49] with an academic license. In all FBA problems, the objective was the wild-type biomass reaction in the most recent *Escherichia coli* metabolic model [50]. The yeast model iMM904 [22] was used for all growth limitation and TEAM simulations, with media formulations matching those described in the original publications.

### Calculation of Temporal Variation in *Escherichia coli*

For all simulations relating to *E. coli*, we used the metabolic network reconstruction iJO1366 [50]. Growth medium compositions for all experiments simulated with the model were obtained from the corresponding experiment references. In all cases, the medium was based on the minimal salts medium [51] with 10 mM ammonium. For experiments from [28], we removed glucose from the media formulations and replaced it with the appropriate limiting carbon source.

In order to calculate temporal variation of metabolite, we use the coefficient of variation (CV):
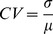
where 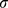 is the standard deviation of the measurements and 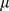 is the mean. For all experiments from both publications, we calculated temporal variation using time points up to 30 minutes following perturbation.

### Permutation Test to Evaluate Significance of Correlation between Shadow Prices and Temporal Variation

In the main text, we show that metabolites with large negative shadow prices exhibit little temporal variation, and metabolites with large temporal variation should exhibit small (or zero) shadow price. To further corroborate the significance of the anticorrelation between shadow prices and temporal variation illustrated in Figure 3, we completed a nonparametric permutation test.

For each experiment, the vector of shadow prices (***λ***) and vector of temporal variation (***CV***) of each metabolite were calculated. Then, the mean temporal variation (*m_T_*) and mean shadow price (*m_S_*) for the experiment were determined using ***λ*** and ***CV***, respectively. We then computed the number of metabolites, *p_original_*, which exhibited a shadow price more negative than *m_S_* and a temporal variation larger than *m_T_*. These metabolites served as a proxy for the number of “incorrect” assignments made by our model.

We generated 10^5^ random permutations of ***λ*** and ***CV***. For each permutation *i*, we calculated *p_i_*, the total number of metabolites satisfying the two criteria described above (exhibited a shadow price more negative than *m_S_* and a temporal variation larger than *m_T_*). Then, we identified the proportion of permutations for which *p_i_<p_original_* (*i.e.* the permuted data exhibited fewer incorrect predictions than the real data), reported in Table S2. We repeated these tests using medians instead of means, with data reported in Table S2.

### Calculation of the Penalty Vector c for TEAM

The penalty vector ***c*** quantifies the modeler's expectation that a reaction is metabolically active (that is, that it carries flux) to an extent that depends on the expression of its constituent genes. The ***c*** vector is calculated by assigning a penalty to each gene in the metabolic model, and then propagating these penalties to the reactions using the Boolean gene-to-reaction mapping provided in the model iMM904 [22]. The higher the value of the penalty *c_i_* for reaction *i*, the higher our confidence that the reaction is inactive. In contrast, reactions with *c* = 0 are expected to be active and carry flux. Importantly, each element of ***c*** is calculated using experimental measurements of gene expression.

First, we describe how we assign a penalty to each gene *g* in the metabolic model. Gene penalties are determined by comparing the expression value of a gene with a predefined threshold. For each gene *g*, we created a cumulative distribution function (CDF) of all expression measurements for that gene (using all gene expression data reported in [32]). Then, for a chosen percentile *θ* (in Figure 5C, we use *θ* = .88), we use the CDF to calculate (for each gene) the expression value corresponding to that percentile. This was the gene's penalty threshold *x_g_*. For the purposes of this article, the primary difference between TEAM [31] and GIMME (an algorithm upon which TEAM is based, see [30]) is that TEAM assigns *unique* penalty thresholds to each gene in the metabolic model, while GIMME assigns a *common* penalty threshold to each gene. In [31], we showed that these gene-specific thresholds substantially increase the accuracy of the algorithm.

Once the gene expression penalty thresholds have been calculated, the penalty for each gene *g*, *p_g_*, is calculated:
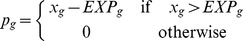
where *EXP_g_* is the expression of gene *g*. Thus, if a gene's expression is above the penalty threshold *x_g_*, that gene is assigned no penalty. In contrast, if its expression is found to be below the threshold, then its penalty is equal to the difference between the two. As described in [31], we used the gene-to-reaction matrix provided in the metabolic model to map the vector of gene penalties *p* to a vector of reaction penalties *c*.

### Calculation of RMF Flux in TEAM

An essential part of TEAM's formulation is a user-defined required metabolic functionality (RMF). The RMF is a metabolic behavior (such as growth or the secretion of a metabolite) that TEAM must reproduce. It was observed in [32] that the population of yeast secreted acetate at the end of the oxidative portion of the metabolic cycle. We recreated *in silico* the environmental conditions of the experiments. We decided to use acetate secretion as our RMF flux. To do so, we first used FBA to identify the maximal amount of acetate that could be secreted by solving the optimization problem
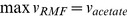
(14)

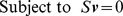


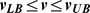
Then, the minimal RMF flux *v_RMF,min_* was set to some proportion *p* of this maximal secretion rate. We used *p = 0.3*, although other values of *p* yielded qualitatively similar results.

### Comparing Time Points between Metabolomics and Transcriptomics Data in TEAM

Because the metabolomics and transcriptomics measurements were obtained from two distinct experiments in which the periods of the cycles were significantly different (∼8 hours vs. ∼12 hours, respectively), we used dissolved oxygen measurements (DO) (which the authors of [32] repeatedly cited as representative of the population's location in the cycle) to align timepoints from the two datasets. The experiments were otherwise comparable in terms of conditions and phenomena observed. All metabolomics data is represented in Figure 5C as fold changes.

### Calculation of Shadow Prices and TEAM Sensitivity Analysis

In order to validate whether the results using TEAM were dependent on our choice of penalty threshold 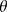, we applied a sensitivity analysis identical to the one described in [31]. We calculated the Spearman correlation for all possible percentile thresholds 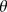 from 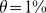 to 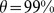 for the same expression and metabolomics time points as those in Figure 5C.

As shown in Figure S1, we found a large range of thresholds for which we obtained high accuracy and a significant correlation, confirming that our results were not highly sensitive to choice of penalty threshold.

## Supporting Information

Dataset S1 **Supporting Data.** Contains data associated with the figures.
(XLSX)

Figure S1 **TEAM's shadow prices are predictive of metabolomics measurements for a large range of penalty thresholds.** We evaluated the sensitivity of the predictive power of TEAM's shadow prices to the particular choice of penalty threshold *θ*. We calculated the Spearman correlation between shadow prices and observed changes in metabolite abundance for *θ* = 1% to *θ* = 99% . Expression data (hour 35 of [32]) and metabolomics data (changes in abundance between hours 10 and 11 in [33]) are identical to those in Figure 5C. For a large part of parameter space we observe significant correlations (p-value<0.05, corresponding to points below dashed line in bottom panel).
(TIF)

Table S1 **Correlations between shadow prices and measures of growth-limitation from** [**21**] **.** For lumped datasets (*e.g.* “All Conditions”) we use non-normalized shadow prices (as in the main part of Figure 1). Note that in the top set of correlations, many metabolites (*e.g.* ATP) exist in several compartments, and the shadow prices in each compartment were used in the statistical test. While in most cases the shadow prices across compartments were identical, there were several instances where this was not the case (see Dataset S1 for data).
(DOCX)

Table S2 **Results of permutation testing of shadow prices and temporal variation.** For all experimental conditions, fewer than 5% of permuted shadow prices exhibited fewer incorrect predictions than the true shadow prices.
(DOCX)

Text S1 **Supporting Text.** Contains additional details for the calculations presented in the text.
(DOCX)
