# Supplementary figures and images for: Flux Imbalance Analysis and the Sensitivity of Cellular Growth to Changes in Metabolite Pools
Source: PLoS Comput Biol. 2013 Aug 29;9(8):e1003195. doi: 10.1371/journal.pcbi.1003195 (PMC3757068; doi:10.1371/journal.pcbi.1003195)

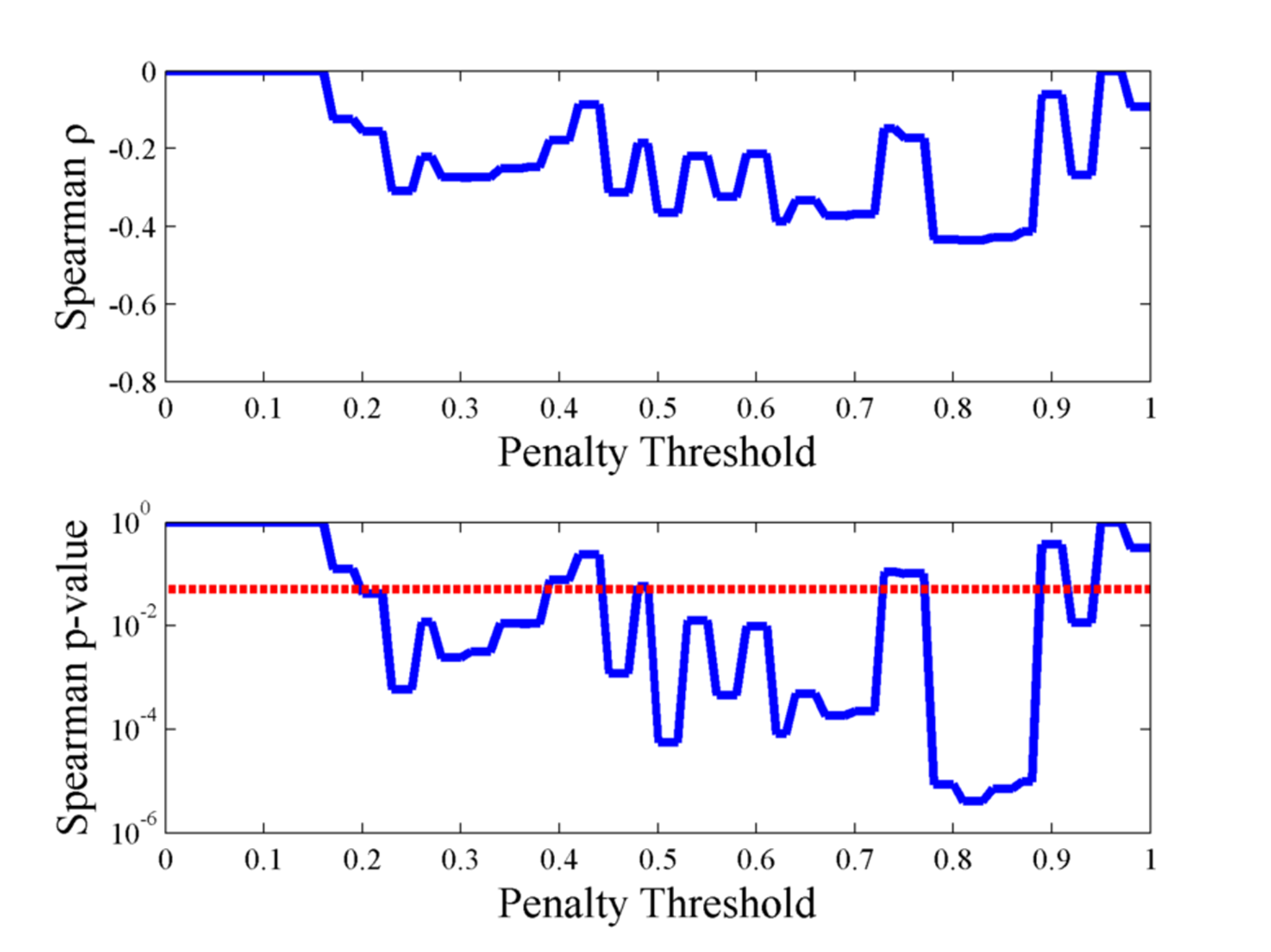

Supplement: Figure S1 — TEAM's shadow prices are predictive of metabolomics measurements for a large range of penalty thresholds. We evaluated the sensitivity of the predictive power of TEAM's shadow prices to the particular choice of penalty threshold θ. We calculated the Spearman correlation between shadow prices and observed changes in metabolite abundance for θ = 1% to θ = 99% . Expression data (hour 35 of [32]) and metabolomics data (changes in abundance between hours 10 and 11 in [33]) are identical to those in Figure 5C. For a large part of parameter space we observe significant correlations (p-value<0.05, corresponding to points below dashed line in bottom panel). (TIF) [file pcbi.1003195.s002.tif]
