# Supplementary material for: Flux Imbalance Analysis and the Sensitivity of Cellular Growth to Changes in Metabolite Pools
Source: PLoS Comput Biol. 2013 Aug 29;9(8):e1003195. doi: 10.1371/journal.pcbi.1003195 (PMC3757068; doi:10.1371/journal.pcbi.1003195)
Supplement: Table S1 — Correlations between shadow prices and measures of growth-limitation from [21] . For lumped datasets (e.g. “All Conditions”) we use non-normalized shadow prices (as in the main part of Figure 1). Note that in the top set of correlations, many metabolites (e.g. ATP) exist in several compartments, and the shadow prices in each compartment were used in the statistical test. While in most cases the shadow prices across compartments were identical, there were several instances where this was not the case (see Dataset S1 for data). (DOCX) [file pcbi.1003195.s003.docx]

**Table S1**

|  | **Spearman** *ρ* | **Spearman p-value** | **Pearson *r*** | **Pearson p-value** | **Number of Data Points** |
| --- | --- | --- | --- | --- | --- |
| **All Conditions** | -0.70 | 2x10^-13^ | -0.28 | 0.006 | 79 |
| **Natural conditions (only Glucose, Nitrogen, and Phosphate Limitation)** | -0.87 | 2x10^-8^ | -0.69 | 8x10^-5^ | 67 |
| **Only Glucose Limitation** | -0.59 | 0.008 | -0.54 | 0.016 | 16 |
| **Only Nitrogen Limitation** | -0.74 | 2x10^-5^ | -0.77 | 1x10^-5^ | 23 |
| **Only Phosphate Limitation** | -0.66 | 5x10^-5^ | -0.50 | 0.033 | 28 |
| **Only Leucine Auxotroph** | 0 | 1 | 0 | 1 | 7 |
| **Only Uracil Auxotroph** | -0.15 | 0.5 | -0.61 | 0.13 | 5 |
| **All Conditions** (Cytosolic Metabolites) | -0.73 | 2x10^-8^ | -0.37 | 0.0075 | 43 |
| **Natural conditions (only Glucose, Nitrogen, and Phosphate Limitation)** (Cytosolic Metabolites) | -0.81 | 7x10^-7^ | -0.23 | 0.14 | 36 |
| **Only Glucose Limitation** (Cytosolic Metabolites) | -0.64 | 0.069 | -0.61 | 0.07 | 7 |
| **Only Nitrogen Limitation** (Cytosolic Metabolites) | -0.83 | 5 x10^-4^ | -0.82 | 5 x10^-4^ | 12 |
| **Only Phosphate Limitation** (Cytosolic Metabolites) | -.70 | 8x10^-4^ | -0.64 | 0.003 | 17 |
| **Only Leucine Auxotroph** (Cytosolic Metabolites) | 0 | 1 | 0 | 1 | 4 |
| **Only Uracil Auxotroph** (Cytosolic Metabolites) | 0 | 0.67 | -0.5 | 0.33 | 3 |
