# Supplementary material for: Flux Imbalance Analysis and the Sensitivity of Cellular Growth to Changes in Metabolite Pools
Source: PLoS Comput Biol. 2013 Aug 29;9(8):e1003195. doi: 10.1371/journal.pcbi.1003195 (PMC3757068; doi:10.1371/journal.pcbi.1003195)
Supplement: Table S2 — Results of permutation testing of shadow prices and temporal variation. For all experimental conditions, fewer than 5% of permuted shadow prices exhibited fewer incorrect predictions than the true shadow prices. (DOCX) [file pcbi.1003195.s004.docx]

**Table S2**

|  | **Proportion of Permutated Datasets with Lower Number of Incorrect Predictions Using Medians** | **Proportion of Permutated Datasets with Lower Number of Incorrect Predictions Using Means** |
| --- | --- | --- |
| **Nitrogen Upshift** | 0.010 | 0.045 |
| **Glucose Starvation** | 0.022 | 0.039 |
| **Acetate Limitation** | 0.001 | 0.0003 |
| **Succinate Limitation** | 0.026 | 0.061 |
| **Glycerol Limitation** | 0.006 | 0.0001 |
