# Supplementary material for: Flux Imbalance Analysis and the Sensitivity of Cellular Growth to Changes in Metabolite Pools
Source: PLoS Comput Biol. 2013 Aug 29;9(8):e1003195. doi: 10.1371/journal.pcbi.1003195 (PMC3757068; doi:10.1371/journal.pcbi.1003195)
Supplement: Text S1 — Supporting Text. Contains additional details for the calculations presented in the text. (DOCX) [file pcbi.1003195.s005.docx]

**C*alculation of Measures of Growth Limitation and Comparison with Shadow Prices***

In this section, we first briefly describe the experiments from reference [1]. Then, we reproduce and elaborate on the theoretical results from [1] which illustrate the connection between changes in metabolite abundance and growth-limitation.

*Description of Experiment*

The experimental growth-limitation of intracellular metabolites was assessed for yeast grown in a chemostat, following [1]. For three different conditions (single nutrient limitation on glucose, nitrogen, and phosphate), and two auxotrophic mutants (leucine and uracil) the abundance of intracellular metabolites was quantified for several different dilution (growth) rates. The authors showed that the growth limitation of a metabolite could be quantified by measuring the change in metabolite abundance at different, increasing growth rates. Metabolites in relatively low abundance which *increased* in abundance as growth rate increased were “growth-limiting.” In contrast, metabolites in relatively high abundance which *decreased* in abundance as growth rate increased were described as “overflow” metabolites, and were *not growth-limiting.*

*A Model for a Quantitative Measure of Growth Limitation (reproduced from* [1] )

For each condition (i.e. each limiting environmental nutrient), the authors in [1] fit the metabolite abundance (*M*) vs. growth rate (*µ*) data to a linear model (where *M_0_* and *µ_0_* are the geometric mean of each metabolite’s concentration measurements)

$\log\left( \frac{M}{M_{0}} \right)=a\log\left( \frac{\mu}{\mu_{0}} \right)+b$ (1)

Here the slope *a* corresponds to the growth-limitation of the metabolite in that particular condition. Based on the derivation presented in [1], and on the classic Monod model for the growth rate of an organism

$\mu=\frac{\mu_{max}M}{K+M}$ (2)

(where *K* is the half-saturation constant), we show here how the slope *a* is related to whether a metabolite is or is not limiting for growth.

It is useful to consider the extreme cases $M\gg K$ (*M* is not growth-limiting) and $M\ll K$ (*M* is very growth-limiting). First, if $M\gg K$ :

$\mu=\frac{\mu_{max}M}{K+M}=\frac{\mu_{max}}{1+\frac{K}{M}}=\mu_{max}\left( 1-\frac{K}{M}+O\left( \left( \frac{K}{M} \right)^{2} \right) \right)$

To first order, we can approximate this expression as

$$\mu\approx\mu_{max}\left( 1-\frac{K}{M} \right)$$

Upon dividing by *µ_max_*, taking the logarithm and using again the $M\gg K$ condition, one can obtain

$$\log\left( \frac{\mu}{\mu_{max}} \right)=\frac{-K}{M}$$

Taking a derivative with respect to the metabolite concentration, yielding:

$$\frac{d\log\left( \frac{\mu}{\mu_{max}} \right)}{dM}=\frac{K}{M^{2}}$$

Upon minor rearrangements this gives:

$$M\frac{d\log\left( \frac{\mu}{\mu_{max}} \right)}{dM}=\frac{d\log\left( \frac{\mu}{\mu_{max}} \right)}{d\log M}=\frac{K}{M}\approx0$$

When *M* is growth-limiting (so that $M\ll K$) the Monod model reduces to

$$\mu\approx\mu_{max}M$$

As before, we can take the logarithm

$$\log\left( \frac{\mu}{\mu_{max}} \right)=\log M$$

And derive with respect to *M*, giving

$$\frac{d\log\left( \frac{\mu}{\mu_{max}} \right)}{d\log M}=1$$

So we expect the slope *a* to be positive. Note that *a* can be greater than one if the dependence of growth-rate on the metabolite obeys a Hill-like function with exponent *n>*1.

*Calculation of the Quantitative Measure of Growth Limitation*

We define *L,* the quantitative measure of growth limitation, to be the slope *a* from equation (1) above. Calculations of *L* were taken from [1]. As in [1], only metabolites which exhibited a significant fit to equation (2) and could be classified as significantly “growth-limiting” or “overflow” metabolites were used.

**References**

1. Boer VM, Crutchfield CA, Bradley PH, Botstein D, Rabinowitz JD (2010) Growth-limiting intracellular metabolites in yeast growing under diverse nutrient limitations. Molecular biology of the cell 21: 198–211. doi:10.1091/mbc.E09-07-0597.

2. Yuan J, Doucette CD, Fowler WU, Feng X-J, Piazza M, et al. (2009) Metabolomics-driven quantitative analysis of ammonia assimilation in E. coli. Molecular systems biology 5: 302. doi:10.1038/msb.2009.60.

3. Xu Y-F, Amador-Noguez D, Reaves ML, Feng X-J, Rabinowitz JD (2012) Ultrasensitive regulation of anapleurosis via allosteric activation of PEP carboxylase. Nature chemical biology 8: 562–568. doi:10.1038/nchembio.941.

4. Tu BP, Kudlicki A, Rowicka M, McKnight SL (2005) Logic of the yeast metabolic cycle: temporal compartmentalization of cellular processes. Science (New York, NY) 310: 1152–1158. doi:10.1126/science.1120499.

5. Tu BP, Mohler RE, Liu JC, Dombek KM, Young ET, et al. (2007) Cyclic changes in metabolic state during the life of a yeast cell. Proceedings of the National Academy of Sciences of the United States of America 104: 16886–16891. doi:10.1073/pnas.0708365104.
